# Nicotine and tobacco product use among US middle and high school students, 2024-25

**DOI:** 10.1186/s12916-026-05046-w

**Published:** 2026-07-07

**Authors:** Haoxiong Sun, Yu Jiang, Harry Tattan-Birch, Siyuan Fan, Sharon Cox, Sarah E. Jackson

**Affiliations:** 1 Department of Behavioural Science and Health, University College London, 1–19 Torrington Place, London, WC1E 7HB UK; 2 School of Public Health, Imperial College London, London, UK

**Keywords:** e-cigarettes, nicotine pouches, youth, tobacco, nicotine

## Abstract

**Background:**

The overall prevalence of youth nicotine and tobacco product use has declined over recent years, but the product landscape continues to change rapidly. This study examined changes between 2024 and 2025 in the prevalence of nicotine and tobacco product use among US middle and high school students and described shifts in product characteristics among current e-cigarette and nicotine pouch users.

**Methods:**

Repeated cross-sectional study using nationally representative data from the 2024 and 2025 National Youth Tobacco Survey (NYTS), a school-based survey of US students in grades 6–12 (approximately ages 11–18). The analytic sample included 29,678 students in 2024 and 23,557 students in 2025. Adjusted prevalence ratios were estimated using survey-weighted modified Poisson regression.

**Results:**

In 2025, 7.2% (95% CI, 6.4–8.2%) of US middle and high school students reported past 30-day use of any nicotine/tobacco product, compared with 8.1% (7.4–8.9%) in 2024; this modest difference was compatible with no change (aPR 0.89; 95% CI 0.77–1.02). E-cigarettes remained the most commonly used product (5.2%, 4.5–5.9%); 1.7% (1.4–2.1%) used nicotine pouches, 1.7% (1.4–1.9%) smoked cigarettes, and 2.7% (2.4–3.1%) smoked any combustible tobacco product. Among current e-cigarette users, 40.7% (36.7–44.9%) reported frequent use and 27.0% (24.0-30.2%) reported daily use in 2025. Disposable e-cigarette use increased from 55.8% (52.6–59.0%) in 2024 to 66.7% (62.5–70.7%) in 2025, while pod/cartridge device use declined. Flavored product use was reported by 90.0% of e-cigarette users and 88.0% of nicotine pouch users. The most commonly reported brands were Geek Bar among e-cigarette users (61.1%) and ZYN among nicotine pouch users (69.4%).

**Conclusions:**

Overall youth nicotine and tobacco use remains relatively low, but the product landscape is being reshaped, with increasing disposable device use and shifting brand preferences. These findings highlight the importance of ongoing, product-specific surveillance to inform public health strategies and regulatory policies.

**Supplementary Information:**

The online version contains supplementary material available at https://doi.org/10.1186/s12916-026-05046-w.

## Background

Although cigarette smoking has decreased steadily among US youth since the mid-1990s [1], adolescent use of nicotine and tobacco products remains an important focus for public health surveillance. These products include cigarettes and other combustible tobacco products, heated tobacco products, and non-combustible products such as e-cigarettes, smokeless tobacco and, more recently, nicotine pouches. The marketplace is evolving rapidly, with changes in product availability, device types, brand popularity, and flavors. Alongside ongoing regulatory actions targeting flavored products and premarket authorization requirements, these shifts underscore the need for continued monitoring to better understand youth nicotine use patterns and inform public health and regulatory responses.

The National Youth Tobacco Survey (NYTS) provides nationally representative data used by the Centers for Disease Control and Prevention (CDC) and the US Food and Drug Administration (FDA) to monitor youth nicotine and tobacco product use in the US [2–6]. These data enable timely assessment of changes in product prevalence as well as characteristics such as frequency of use, device type, brands, and flavors among youth users. Although NYTS is US-specific, the scale of the US market means even low prevalence represents large numbers of users, allowing dominant brands to gain market share and international marketing influence, and monitoring these dynamics offers a signal for other countries facing similar shifts. The primary aim of this study was to examine changes between 2024 and 2025 in the prevalence of nicotine and tobacco product use among US middle and high school students. A secondary aim was to describe changes in frequency of use, brand, device type, and flavor preferences among current e-cigarette and nicotine pouch users.

## Methods

### Study design and participants

This repeated cross-sectional study used publicly available, de-identified data from the 2024 and 2025 National Youth Tobacco Survey (NYTS) and therefore did not require institutional review board approval. The study followed the Strengthening the Reporting of Observational Studies in Epidemiology (STROBE) reporting guideline for cross-sectional studies [7].

The NYTS is a school-based, self-administered, web-based survey of US middle school (grades 6–8) and high school (grades 9–12) students (approximately ages 11–18) conducted by the Centers for Disease Control and Prevention and the US Food and Drug Administration [8]. The survey uses a stratified, three-stage cluster sampling design to generate nationally representative estimates for students attending public and private schools in the 50 states and the District of Columbia [8].

Data were collected during January through May in both survey years. In 2024, 29,861 students from 283 schools participated, with an overall response rate of 33.4% [4]. In 2025, 23,630 students participated, with a response rate of 29.7% [8]. After excluding respondents with missing information on their school level, the analytic sample comprised 29,678 students in 2024 and 23,557 students in 2025.

### Measures

Full details of survey measures are provided in the NYTS methodology report [8, 9]. Key measures are summarized below.

#### Nicotine/tobacco product use

Past 30-day (current) use was defined as use on 1 or more days during the 30 days preceding the survey. Current use was assessed for 12 nicotine/tobacco products: e-cigarettes, nicotine pouches, manufactured cigarettes, roll-your-own cigarettes, cigars (including cigarillos and little cigars), smokeless tobacco (including chewing tobacco, snuff, and dip), snus, hookahs, heated tobacco products, oral nicotine products (including lozenges, discs, tablets, gums, and dissolvable tobacco products), pipe tobacco, and bidis. Nicotine pouches were analysed separately from other oral nicotine products, consistent with the NYTS questionnaire structure and NYTS publications [4, 10].

Three composite measures were constructed: [1] any nicotine/tobacco product, defined as current use of at least 1 of the 12 products; [2] any combustible tobacco product, defined as current use of manufactured cigarettes, roll-your-own cigarettes, cigars, hookahs, pipe tobacco, or bidis; and [3] cigarettes, defined as current use of manufactured or roll-your-own cigarettes.

#### E-cigarette and nicotine pouch use characteristics

Students who currently use e-cigarettes or nicotine pouches reported their frequency of use (number of days used in the past 30 days), brands used, and flavors used. Four categories were created to characterize frequency of use: occasional (1–5 days), moderate (6–19 days), frequent (≥ 20 days), and daily (all 30 days) [10]. Current e-cigarette users also reported the device type they used most often (disposable, pod or cartridge-based, or tank or mod system).

For check-all-that-apply brand and flavor items, each response option was coded as a separate binary indicator. Two composite flavor variables were created for both e-cigarettes and nicotine pouches: mint/menthol (any reported use of mint or menthol) and any flavored (use of at least 1 flavor other than tobacco or unflavored). Because some brand response options differed between survey years, cross-year brand comparisons were interpreted cautiously.

#### Demographic characteristics

Demographic characteristics included sex (female, male), school level (middle school, high school), and race and ethnicity. Race/ethnicity were self-reported and categorized as Hispanic, non-Hispanic American Indian or Alaska Native, non-Hispanic Asian, non-Hispanic Black, non-Hispanic White, and non-Hispanic multiracial or other.

### Statistical analysis

All analyses accounted for the NYTS complex survey design by incorporating primary sampling units, strata, and final analytic weights to produce nationally representative estimates and appropriate variance estimates.

For the 2024 and 2025 middle school subgroup, two variance estimation strata contained singleton or near-singleton primary sampling units, producing non-estimable confidence intervals (CIs). To address this, adjacent strata were collapsed before variance estimation, and sensitivity analyses using alternative variance estimation methods confirmed the robustness of these estimates in Additional file 1: Table S1 [11, 12].

Weighted prevalence estimates and 95% CIs were calculated for past-30-day (current) use of each product by year, overall and stratified by school level and sex (sex-stratified results are provided in Additional file 1: Table S2). CIs were estimated using the logit transformation method. National estimated numbers of users were calculated from survey-weighted population totals and rounded to the nearest 10,000.

Among current e-cigarette users and nicotine pouch users, weighted proportions and 95% confidence intervals were estimated for frequency of use, device type, brands, and flavors. Year-to-year differences in weighted proportions were assessed using design-based Wald tests. For comparisons of use characteristics among current users, unadjusted differences in weighted proportions and corresponding Wald test P values are reported, as these analyses describe within-user characteristics rather than population-level prevalence.

Changes in prevalence between 2024 and 2025 were assessed using Wald tests based on the pooled standard error of the difference in weighted proportions. Adjusted prevalence ratios (aPRs) and 95% confidence intervals were estimated using survey-weighted modified Poisson regression with a log link and design-based variance estimation. In overall models, the main exposure was survey year (2025 vs. 2024), and models adjusted for school level, sex, and race/ethnicity. School-level subgroup models adjusted for sex and race/ethnicity.

All statistical tests were 2-sided. For each analysis, respondents with missing data on the outcome or model covariates were excluded from that specific analysis (complete-case analysis), as item missingness on all outcome and covariate variables was low (< 5%; Additional file 1: Table S3). Analyses were conducted in R, version 4.5.2 (R Foundation for Statistical Computing).

## Results

### Past 30-day use of any nicotine/tobacco product

In 2025, 7.2% of US middle and high school students reported past-30-day use of any nicotine/tobacco product, corresponding to approximately 2.0 million students, compared with 8.1% in 2024 (~ 2.23 million) (Table 1). Adjusted analyses indicated that prevalence was modestly lower in 2025 than in 2024, although this difference was small and compatible with no change (aPR 0.89; 95% CI 0.77–1.02).

By school level, 4.2% of middle school students and 9.5% of high school students reported past-30-day use of any product in 2025. Adjusted prevalence was lower in 2025 than 2024 among middle school students (aPR 0.76; 95% CI 0.60–0.96), while the change among high school students was smaller and the confidence interval included the null (aPR 0.93; 95% CI 0.80–1.09).

### Types of products used

E-cigarettes remained the most commonly used nicotine/tobacco product, while nicotine pouches and combustible tobacco were less prevalent (Table 1).

Overall, past 30-day e-cigarette use was 5.2% in 2025 (~ 1.43 million students), down from 5.9% in 2024 (~ 1.62 million). In 2025, prevalence was 2.6% among middle school students and 7.1% among high school students. Adjusted prevalence was lower in 2025 among middle school students (aPR 0.72; 95% CI 0.55–0.94), while among high school students the change was modest and compatible with no difference (aPR 0.92; 95% CI 0.78–1.09).

Past 30-day nicotine pouch use was 1.7% in 2025 (~ 450,000 students), similar to 1.8% in 2024 (~ 470,000). In 2025, prevalence was 0.9% among middle school students and 2.3% among high school students, with prevalence similar across years overall and by school level. Prevalence of nicotine pouch use remained higher among boys (2.4%) than girls (1.0%; Additional file 1: Table S2).

Among other non-combustible products, smokeless tobacco use declined modestly overall from 0.9% in 2024 to 0.7% in 2025 (aPR 0.73; 95% CI, 0.55–0.97), whereas oral nicotine product use declined more markedly from 1.2% to 0.6% (aPR 0.52; 95% CI, 0.42–0.64). Oral nicotine product use was lower in 2025 in both middle school students (0.9% to 0.5%; aPR 0.60; 95% CI 0.41–0.88) and high school students (1.4% to 0.7%; aPR 0.47; 95% CI 0.36–0.62).

Past 30-day use of any combustible tobacco was 2.7% in 2025, compared with 3.0% in 2024. In 2025, prevalence was 1.9% among middle school students and 3.4% among high school students. Cigarettes, including roll-your-own cigarettes, were the most commonly reported combustible product, with prevalence of 1.7% overall; 1.1% among middle school students and 2.1% among high school students. Among other combustible products, cigars (1.0%), hookah (0.7%), pipe tobacco (0.4%), and bidis (0.4%) were each used by no more than 1% of students in 2025, with prevalence similar to 2024. Heated tobacco products (0.6%) and snus (0.6%) also remained uncommon in 2025, with prevalence comparable across years. Complete prevalence estimates for all individual products are presented in Table 1, with sex-specific estimates in Additional file 1: Table S2.

**Table 1:** Estimated Prevalence of Past 30-Day Nicotine/Tobacco Product Use among US Middle and High School Students, 2024–2025

Product / School Level	2024% (95% CI)	2025% (95% CI)	aPR (95% CI)	*P*-value^a^
**Any Nicotine/Tobacco Product ** ^c^				
Overall	8.1 (7.4–8.9)	7.2 (6.4–8.2)	0.89 (0.77–1.02)	0.083
Middle School	5.5 (4.7–6.4)	4.2 (3.5–5.2) ^**b**^	0.76 (0.60–0.96)	0.021
High School	10.1 (9.0-11.3)	9.5 (8.4–10.7)	0.93 (0.80–1.09)	0.387
**E-cigarettes**				
Overall	5.9 (5.3–6.6)	5.2 (4.5–5.9)	0.87 (0.75–1.01)	0.067
Middle School	3.5 (2.9–4.2)	2.6 (2.1–3.2) ^**b**^	0.72 (0.55–0.94)	0.017
High School	7.8 (6.9–8.8)	7.1 (6.3–8.1)	0.92 (0.78–1.09)	0.317
**Nicotine Pouches**				
Overall	1.8 (1.5–2.1)	1.7 (1.4–2.1)	0.94 (0.75–1.19)	0.617
Middle School	1.0 (0.8–1.2)	0.9 (0.7–1.2) ^**b**^	0.94 (0.68–1.31)	0.712
High School	2.4 (2.0-2.9)	2.3 (1.8–2.9)	0.94 (0.71–1.23)	0.631
**Any Combustible Tobacco** ^**d**^				
Overall	3.0 (2.6–3.3)	2.7 (2.4–3.1)	0.93 (0.78–1.10)	0.381
Middle School	2.2 (1.8–2.8)	1.9 (1.4–2.4) ^**b**^	0.81 (0.59–1.12)	0.207
High School	3.5 (3.0-4.1)	3.4 (2.9–3.8)	0.98 (0.81–1.18)	0.802
**Cigarettes (including RYO)** ^**e**^				
Overall	1.8 (1.6–2.1)	1.7 (1.4–1.9)	0.93 (0.77–1.13)	0.471
Middle School	1.4 (1.2–1.8)	1.1 (0.8–1.5) ^**b**^	0.76 (0.54–1.07)	0.122
High School	2.1 (1.7–2.4)	2.1 (1.7–2.4)	1.01 (0.81–1.27)	0.906
**Manufactured Cigarettes Only**				
Overall	1.4 (1.2–1.6)	1.4 (1.2–1.6)	0.99 (0.81–1.22)	0.953
Middle School	1.1 (0.8–1.4)	0.8 (0.6–1.1) ^**b**^	0.77 (0.53–1.13)	0.182
High School	1.7 (1.4-2.0)	1.8 (1.5–2.2)	1.09 (0.86–1.39)	0.466
**Roll-Your-Own Cigarettes**				
Overall	0.6 (0.5–0.8)	0.5 (0.4–0.6)	0.80 (0.60–1.06)	0.116
Middle School	0.6 (0.5–0.8)	0.5 (0.3–0.7) ^**b**^	0.75 (0.50–1.12)	0.159
High School	0.7 (0.5–0.9)	0.5 (0.4–0.7)	0.84 (0.58–1.22)	0.361
**Cigars**				
Overall	1.2 (1.0-1.4)	1.0 (0.8–1.3)	0.88 (0.67–1.16)	0.362
Middle School	0.8 (0.6-1.0)	0.8 (0.6–1.1) ^**b**^	1.05 (0.69–1.61)	0.817
High School	1.5 (1.2–1.9)	1.2 (0.9–1.6)	0.81 (0.57–1.14)	0.227
**Hookah**				
Overall	0.7 (0.6–0.9)	0.7 (0.5–0.8)	0.96 (0.72–1.28)	0.773
Middle School	0.6 (0.4–0.8)	0.5 (0.4–0.8) ^**b**^	0.92 (0.57–1.47)	0.726
High School	0.8 (0.6–1.1)	0.8 (0.6-1.0)	0.98 (0.68–1.41)	0.907
**Pipe Tobacco**				
Overall	0.5 (0.4–0.6)	0.4 (0.3–0.5)	0.74 (0.53–1.03)	0.074
Middle School	0.5 (0.3–0.7)	0.3 (0.2–0.4) ^**b**^	0.57 (0.32–1.01)	0.054
High School	0.5 (0.4–0.6)	0.4 (0.3–0.6)	0.86 (0.56–1.32)	0.494
**Bidis**				
Overall	0.5 (0.4–0.6)	0.4 (0.3–0.6)	0.88 (0.65–1.18)	0.391
Middle School	0.5 (0.3–0.7)	0.4 (0.3–0.6) ^**b**^	0.82 (0.51–1.29)	0.386
High School	0.5 (0.4–0.7)	0.5 (0.3–0.7)	0.92 (0.62–1.37)	0.679
**Smokeless Tobacco**				
Overall	0.9 (0.7–1.1)	0.7 (0.5–0.9)	0.73 (0.55–0.97)	0.028
Middle School	0.7 (0.5–0.9)	0.5 (0.3–0.7) ^**b**^	0.66 (0.42–1.03)	0.065
High School	1.0 (0.8–1.3)	0.8 (0.6–1.1)	0.73 (0.55–0.97)	0.028
**Snus**				
Overall	0.7 (0.6–0.9)	0.6 (0.4–0.7)	0.80 (0.59–1.08)	0.149
Middle School	0.4 (0.3–0.5)	0.3 (0.2–0.5) ^**b**^	0.83 (0.46–1.49)	0.528
High School	1.0 (0.8–1.2)	0.8 (0.6-1.0)	0.79 (0.57–1.10)	0.160
**Oral Nicotine Products**				
Overall	1.2 (1.0-1.4)	0.6 (0.5–0.8)	0.52 (0.42–0.64)	< 0.001
Middle School	0.9 (0.7–1.1)	0.5 (0.4–0.7) ^**b**^	0.60 (0.41–0.88)	0.009
High School	1.4 (1.2–1.7)	0.7 (0.5–0.9)	0.47 (0.36–0.62)	< 0.001
**Heated Tobacco Products**				
Overall	0.8 (0.7-1.0)	0.6 (0.5–0.8)	0.81 (0.63–1.04)	0.095
Middle School	0.7 (0.6-1.0)	0.6 (0.5–0.9) ^**b**^	0.90 (0.61–1.32)	0.590
High School	0.9 (0.7–1.1)	0.6 (0.5–0.8)	0.75 (0.54–1.04)	0.086

**Abbreviations**: aPR = Adjusted Prevalence Ratio; CI = Confidence Interval; RYO = Roll-Your-Own

^a^ Boldface indicates *P* < 0.05

^b^ Calculated using Taylor linearization with collapsed strata

^c^ Any nicotine/tobacco product includes e-cigarettes, nicotine pouches, oral nicotine products, cigarettes, roll-your-own cigarettes, cigars, smokeless tobacco, snus, hookah, heated tobacco products, pipe tobacco, and bidis

^d^ Any combustible tobacco includes cigarettes, roll-your-own cigarettes, cigars, hookah, pipe tobacco, and bidis

^e^ Cigarettes (including RYO) includes manufactured cigarettes and roll-your-own cigarettes

### E-cigarette use characteristics

In 2025, 40.7% of past 30-day e-cigarette users reported frequent use (≥ 20 of the past 30 days) and 27.0% reported daily use, which were comparable to 2024 (Table 2).

Device preferences shifted notably between survey years. Use of disposable e-cigarettes increased from 55.8% in 2024 to 66.7% in 2025 (*P* < 0.001), while use of pod/cartridge-based devices declined from 15.6% to 11.7%) (*P* = 0.022).

Brand preferences also shifted. In 2025, Geek Bar was the most commonly reported brand (61.1%), however, this should be interpreted with caution as Geek Bar was not included as a prespecified response option in 2024. Elf Bar/EBCreate declined from 36.0% in 2024 to 20.9% in 2025 (*P* < 0.001). Other popular brands in 2025 included Lost Mary (18.5%), Vuse (15.2%), Breeze (13.8%), and JUUL (9.1%).

Flavored e-cigarette use remained high, with 90.0% of users reporting any flavored product in 2025, unchanged from 2024. In contrast, unflavored e-cigarette use declined from 12.1% in 2024 to 8.8% in 2025 (*P* = 0.038). Fruit remained the most commonly used flavor (63.6%), followed by ice/iced (53.0%), mint/menthol (38.8%), and candy/sweets (33.6%) in 2025. Concept or abstract flavors were less frequently used (18.2%). Tobacco-flavored e-cigarette use was reported by 8.5% of users, comparable to 2024.

**Table 2:** Characteristics and Preferences among Past 30-Day E-Cigarette Users, 2024–2025

Characteristic	2024% (95% CI)	2025% (95% CI)	Change (pp)	*P*-value ^a^
Frequency of Use				
Occasional (1–5 days)	44.2 (40.2–48.2)	42.7 (38.8–46.6)	-1.5	0.594
Moderate (6–19 days)	17.6 (15.5–19.6)	16.6 (14.5–18.6)	-1.0	0.499
Frequent (≥ 20 days)	38.2 (34.3–42.2)	40.7 (36.7–44.9)	+ 2.5	0.383
Daily (30 days)	26.1 (22.8–29.7)	27.0 (24.0-30.2)	+ 0.9	0.704
**Device Type**				
Disposable	55.8 (52.6–59.0)	66.7 (62.5–70.7)	+ 10.9	**< 0.001**
Pods / Cartridges	15.6 (13.5–18.0)	11.7 (9.4–14.4)	-3.9	0.025
Tank/Mod	6.9 (5.4–8.4)	5.1 (3.6–6.5)	-1.8	0.082
**Top Flavors**				
Any Flavored	90.0 (88.4–91.6)	90.0 (87.5–92.4)	-0.1	0.973
Fruit	64.6 (61.7–67.5)	63.6 (58.8–68.3)	-1.0	0.728
Ice/Iced	54.5 (51.4–57.7)	53.0 (48.3–57.7)	-1.5	0.592
Mint/Menthol	34.4 (30.9–38.0)	38.8 (34.7–43.0)	+ 4.4	0.116
Candy/Sweets	34.5 (31.4–37.5)	33.6 (30.3–36.9)	-0.9	0.698
Concept/Abstract	20.3 (18.2–22.4)	18.2 (15.7–20.7)	-2.1	0.209
Non-alcoholic Drink	12.1 (10.3–14.1)	11.9 (9.9–14.3)	-0.2	0.898
**Unflavored**	**12.1 (10.1–14.3)**	**8.8 (6.7–11.4)**	**-3.3**	**0.038**
Alcoholic Drink	9.2 (7.4–11.4)	9.1 (7.2–11.5)	-0.1	0.934
Tobacco	8.9 (7.3–10.8)	8.5 (6.6–11.0)	-0.4	0.786
Spice/Herb	6.6 (5.4–8.1)	6.6 (5.0-8.7)	+ 0.0	0.991
Chocolate/Dessert	5.9 (4.5–7.6)	5.4 (3.9–7.4)	-0.5	0.685
**Top Brands**				
Geek Bar ^**c**^	5.8 (4.3–7.8)	61.1 (56.8–65.3)	—	—
Elf Bar / EBCreate	36.0 (32.6–39.5)	20.9 (17.5–24.4)	-15.1	**< 0.001**
Lost Mary ^**c**^	3.4 (2.4–4.9)	18.5 (15.0-22.5)	—	—
Vuse	13.4 (10.3–16.6)	15.2 (10.7–19.8)	+ 1.8	0.524
Breeze	19.6 (14.5–24.6)	13.8 (9.5–18.1)	-5.8	0.088
Mr Fog	15.5 (8.1–22.9)	10.0 (6.1–13.8)	-5.5	0.195
JUUL	12.4 (10.3–14.5)	9.1 (6.4–11.8)	-3.2	0.062
Fume	8.8 (6.7–10.9)	6.3 (4.2–8.5)	-2.4	0.109
NJOY	5.8 (4.4–7.3)	5.7 (3.9–7.6)	-0.1	0.938
Raz ᵇ	—	18.8 (14.8–23.6)	—	—
Juicy Bar ᵇ	—	10.9 (8.6–13.8)	—	—
Kangvape	7.3 (5.6–9.5)	6.4 (4.7–8.5)	-1.0	0.482
blu	6.6 (5.4–8.1)	6.0 (4.5-8.0)	-0.6	0.579
HQD	4.7 (3.6-6.0)	4.4 (3.2–6.2)	-0.2	0.799
Loon ᵇ	—	4.6 (3.1–6.8)	—	—

^**a**^ Boldface indicates *P* < 0.05

^b^Brand was not included as a prespecified response option in 2024

^**c**^ Geek Bar and Lost Mary were not included in 2024, in the list of prespecified response options but were the two most common write-in responses for “some other brand(s) not listed here, based on the MMWR report. The value for 2024 was extracted from previous report directly, used for reference only (10)

Brand and flavor use was assessed using check-all-that-apply items; respondents could select multiple brands

**Abbreviations**: CI = Confidence Interval; pp = percentage points

### Nicotine pouch use characteristics

In 2025, 26.0% of past 30-day nicotine pouch users reported frequent use (≥ 20 of the past 30 days) and 17.3% reported daily use, both lower than in 2024 (29.3% and 22.3%, respectively), although the confidence intervals for the changes were wide and included no difference (Table 3).

**Table 3:** Characteristics and Preferences among Past 30-Day Nicotine Pouch Users, 2024–2025

Characteristic	2024% (95% CI)	2025% (95% CI)	Change (pp)	*P*-value ^a^
Frequency of Use				
Occasional (1–5 days)	53.9 (48.0-59.8)	53.0 (44.9–61.1)	-0.9	0.858
Moderate (6–19 days)	16.8 (12.7–21.0)	21.0 (14.5–27.5)	4.1	0.292
Frequent (≥ 20 days)	29.3 (24.6–34.4)	26.0 (22.0-30.5)	-3.2	0.325
Daily (30 days)	22.3 (18.3–27.0)	17.3 (14.0-21.2)	-5	0.081
**Top Flavors**				
Any Flavored	83.6 (79.6–87.6)	88.0 (84.2–91.8)	+ 4.5	0.114
Mint/Menthol	64.7 (58.7–70.7)	70.3 (64.9–75.7)	5.6	0.172
Fruit	23.5 (18.4–28.6)	25.8 (19.6–31.9)	2.3	0.574
Ice/Iced	23.2 (19.5–27.0)	17.9 (13.3–22.6)	-5.3	0.084
Concept/Abstract	11.5 (8.1–15.0)	11.2 (7.7–14.7)	-0.3	0.896
Candy/Sweets	10.4 (7.7–13.0)	8.6 (5.1–12.1)	-1.8	0.438
Spice/Herb	11.1 (8.1–15.0)	10.3 (7.2–14.6)	-0.7	0.770
Tobacco	8.7 (6.5–11.7)	4.7 (3.1-7.0)	-4.1	0.012
Chocolate/Dessert	8.4 (6.1–11.5)	5.7 (3.1–10.0)	-2.8	0.199
Non-alcoholic Drink	7.7 (5.4–10.8)	9.7 (6.1–15.3)	+ 2.0	0.443
Alcoholic Drink	6.8 (4.6–10.0)	5.3 (3.4–8.1)	-1.6	0.374
Unflavored	14.3 (10.7–18.9)	6.4 (3.9–10.2)	-8.0	0.002
**Top Brands**				
ZYN	68.3 (62.3–74.2)	69.4 (62.5–76.3)	1.1	0.808
on!	14.1 (10.6–17.5)	14.4 (10.7–18.1)	0.3	0.894
VELO	10.6 (7.8–13.3)	12.4 (3.9–20.9)	1.9	0.684
Rogue	13.5 (10.0-16.9)	12.3 (9.1–15.5)	-1.2	0.625
Juice Head	9.6 (7.1–12.0)	7.3 (3.8–10.8)	-2.3	0.295
FRE	9.3 (6.4–12.3)	7.1 (3.9–10.2)	-2.3	0.299
2ONE/ZONE ᶜ	7.0 (5.0-9.8)	10.5 (6.0-17.8)	+ 3.5	0.266
Grizzly ᵇ	—	9.2 (6.5–12.8)	—	—
Black Buffalo ᵇ	—	4.0 (2.5–6.4)	—	—
ZEO ᵇ	—	3.9 (2.0-7.6)	—	—

^a^Boldface indicates P < 0.05

^b^Brand was not included as a prespecified response option in 2024

^c^Listed as "2ONE" in 2024 and "ZONE" in 2025; treated as the same brand

Brand and flavor use was assessed using check-all-that-apply items; respondents could select multiple brands

Abbreviations: CI = Confidence Interval; pp = percentage points

Flavored nicotine pouch use remained high, reported by 88.0% of users in 2025, compared with 83.6% in 2024. In contrast, unflavored pouch use declined from 14.3% in 2024 to 6.4% in 2025 (*P* = 0.002). Mint/menthol was the most commonly used flavor (70.3%), followed by fruit (25.8%). Other flavors, including ice/iced, candy/sweets, and concept/abstract, were less frequently used. Use of tobacco-flavored pouches also declined, from 8.7% in 2024 to 4.7% in 2025 (*P* = 0.012).

Brand preferences were relatively stable. ZYN remained the dominant brand at 69.4% in 2025, similar to 68.3% in 2024. Other brands were reported by smaller proportions of users, with no notable changes between survey years

## Discussion

In this repeated cross-sectional study of nationally representative US middle and high school students, overall past 30-day nicotine and tobacco product use was slightly lower in 2025 than 2024, continuing the downward trajectory observed since 2019, when 23.0% of US middle and high school students reported past 30-day use of any tobacco product [4, 13, 14]. Cigarette smoking prevalence remained at 1.7%, and overall combustible tobacco use remained low at 2.7%. Although prevalence is historically low, this still represents around 2 million adolescent users in 2025, many of whom report frequent or daily use, indicating continued dependence risk. E-cigarettes remained the most commonly used product, with the continued growth of disposable e-cigarettes, shown by the rise of Geek Bar as the dominant brand. Nicotine pouch use was stable at approximately 1.7%, with ZYN reported by nearly 70% of pouch users.

The dominance of Geek Bar among e-cigarette users in 2025, alongside the decline of Elf Bar/EBCreate, illustrates the dynamic reorganization of the youth disposable e-cigarette market. Shifts in brand popularity did not reduce overall e-cigarette use, highlighting how youth may readily switch between products rather than stop using nicotine. The continued growth of disposable e-cigarettes is consistent with international trends. In England, the rapid rise of disposable devices was associated with increases in vaping among young adults, including those who had never regularly smoked [15, 16]. These concerns, along with environmental ones, prompted the United Kingdom to legislate a ban on disposable vapes, with similar bans introduced in Australia and New Zealand [17–19]. When the United Kingdom legislated a ban on disposable vapes, the proportion of vapers using disposables declined, but young adult vapers (aged 16–24) shifted to refillable and rechargeable devices rather than stopping [20]. This pattern of intra-category switching, in which users switch between device formats or brands rather than cease use, is consistent with the brand-switching observed in our US data, and suggests that the impact of product-specific regulations on overall nicotine use among youth requires careful monitoring.

The ongoing decline in combustible tobacco use among youth represents a sustained public health achievement. Cigarette prevalence remained at 1.7%, the lowest level ever recorded by the NYTS, and other combustible products, including cigars, hookah, and pipe tobacco, were each used by no more than 1% of students, with prevalence comparable across years [4]. In relation to newer products, prevalence of nicotine pouch use remained stable across 2024 and 2025, following a period of growth from 0.8% in 2021 to 1.8% in 2024, while the number of users who had never used other tobacco or nicotine products increased substantially [10, 21]. Although overall pouch prevalence was stable, frequency of use among current pouch users showed a directional decline, warranting continued monitoring. The composition of pouch use has shifted: unflavored or tobacco-flavored pouch use declined while flavored use, predominantly mint or menthol, remained high. Although cross-sectional data cannot distinguish whether stable prevalence reflects continued use by the same individuals or turnover in the user population, a key question is whether pouches are substituting for more harmful tobacco products or instead attracting young people who would otherwise not use nicotine [22]. This distinction has important implications for the regulation of oral nicotine pouches. By contrast, use of other oral nicotine products (lozenges, gums, and dissolvable products) declined notably from 1.2% to 0.6%, consistent with broader marketplace trends showing that the growth of pouches has coincided with declines in sales of earlier-generation oral tobacco and nicotine products [23].

Declines in nicotine and tobacco use were concentrated among middle school students for both any tobacco product use and e-cigarette use, while high school prevalence showed less evidence of change. This age-differential pattern is consistent with the possibility that existing tobacco control measures, such as minimum legal sales age requirements and school-based prevention campaigns, may be more effective at deterring initiation among younger adolescents [4, 24, 25]. It may also reflect that high school prevalence has already fallen substantially from earlier peaks [4] or that the available data were insufficient to detect a change within broader influences on youth behavior, as the confidence interval included the possibility of notable year-on-year variation.

This study has several limitations. First, the 2025 NYTS response rate was 29.7%, lower than the 33.4% in 2024, which may affect the representativeness of estimates despite the application of survey weights [4, 8]. Second, cross-year comparisons of brand prevalence should be interpreted cautiously, as several brands, like Geek Bar and Lost Mary, were not included as prespecified response options in 2024 [10]. Third, all measures were self-reported and may be subject to recall or social desirability bias. Fourth, complete-case analysis was used, and missingness on some variables may have introduced bias if data were not missing completely at random. Fifth, although race and ethnicity were included as adjustment variables, a dedicated analysis of disparities across subgroups was beyond the scope of this population-level year-to-year comparison; differential patterns by race and ethnicity should be investigated in future studies. Finally, the NYTS does not capture the timing or sequence of initiation, limiting the ability to characterize use among young people with no prior nicotine experience.

## Conclusion

These findings indicate that although prevalence continues to decline, youth nicotine and tobacco use remains substantial in absolute terms, and the product landscape is shifting rapidly. The speed of brand turnover in the e-cigarette market highlights the challenges of regulating these dynamic markets, where device-level standards and flavour restrictions are likely to be more durable than brand-specific measures. The consolidation of flavored nicotine pouch use, alongside declines in use of unflavored and tobacco-flavored products, requires further investigation into whether the composition of the youth user base is changing over time. Continued product-specific surveillance will be essential to inform timely regulatory and public health responses to these rapidly changing markets.

## Supplementary Information

Below is the link to the electronic supplementary material.

Additional File 1:Table S1-S3

## Data Availability

The data analyzed in this study are publicly available, de-identified microdata from the 2024 and 2025 National Youth Tobacco Survey (NYTS), published by the US Food and Drug Administration’s Center for Tobacco Products. Datasets, codebooks, and methodology reports can be downloaded at https://www.fda.gov/tobacco-products/youth-and-tobacco/national-youth-tobacco-survey-nyts. Analytic code is available from the corresponding author upon reasonable request.

## Abbreviations

aPR: Adjusted Prevalence Ratio
CDC: Centers for Disease Control and Prevention
CI: Confidence Interval
FDA: US Food and Drug Administration
MMWR: Morbidity and Mortality Weekly Report
NYTS: National Youth Tobacco Survey
pp: percentage points
RYO: Roll-Your-Own (cigarettes)
